# Repertoire-wide gene structure analyses: a case study comparing automatically predicted and manually annotated gene models

**DOI:** 10.1186/s12864-019-6064-8

**Published:** 2019-10-17

**Authors:** Jeanne Wilbrandt, Bernhard Misof, Kristen A. Panfilio, Oliver Niehuis

**Affiliations:** 1 Center for molecular Biodiversity Research, Zoological Research Museum Alexander Koenig (ZFMK), Adenauerallee 160, 53113 Bonn, Germany; 2 Present address: Hoffmann Research Group, Leibniz Institute on Aging – Fritz Lipmann Institute, Beutenbergstraße 11, 07745 Jena, Germany; 3 School of Life Sciences, University of Warwick, Gibbet Hill Campus, Coventry, CV4 7AL UK; 4 Evolutionary Biology and Ecology, Institute of Biology I (Zoology), Albert Ludwig University, Hauptstr. 1, 79104 Freiburg, Germany

**Keywords:** Gene prediction, structural annotation, manual annotation, manual curation, exon-intron structure, insects

## Abstract

**Background:**

The location and modular structure of eukaryotic protein-coding genes in genomic sequences can be automatically predicted by gene annotation algorithms. These predictions are often used for comparative studies on gene structure, gene repertoires, and genome evolution. However, automatic annotation algorithms do not yet correctly identify all genes within a genome, and manual annotation is often necessary to obtain accurate gene models and gene sets. As manual annotation is time-consuming, only a fraction of the gene models in a genome is typically manually annotated, and this fraction often differs between species. To assess the impact of manual annotation efforts on genome-wide analyses of gene structural properties, we compared the structural properties of protein-coding genes in seven diverse insect species sequenced by the i5k initiative.

**Results:**

Our results show that the subset of genes chosen for manual annotation by a research community (3.5–7% of gene models) may have structural properties (e.g., lengths and exon counts) that are not necessarily representative for a species’ gene set as a whole. Nonetheless, the structural properties of automatically generated gene models are only altered marginally (if at all) through manual annotation. Major correlative trends, for example a negative correlation between genome size and exonic proportion, can be inferred from either the automatically predicted or manually annotated gene models alike. Vice versa, some previously reported trends did not appear in either the automatic or manually annotated gene sets, pointing towards insect-specific gene structural peculiarities.

**Conclusions:**

In our analysis of gene structural properties, automatically predicted gene models proved to be sufficiently reliable to recover the same gene-repertoire-wide correlative trends that we found when focusing on manually annotated gene models only. We acknowledge that analyses on the individual gene level clearly benefit from manual curation. However, as genome sequencing and annotation projects often differ in the extent of their manual annotation and curation efforts, our results indicate that comparative studies analyzing gene structural properties in these genomes can nonetheless be justifiable and informative.

**Electronic supplementary material:**

The online version of this article (10.1186/s12864-019-6064-8) contains supplementary material, which is available to authorized users.

## Background

Eukaryotic protein-coding gene structure is characterized by a modular organization of introns and exons (the latter being composed of coding sequence [CDS] and/or untranslated regions [UTRs]; [1]), which are commonly identified (with the notable exception of UTRs) in genome sequences using automated in silico gene annotation procedures [2]. The configuration of exons and introns — GC content, length, and number — varies among species, as well as by gene type. A major goal in the field of comparative genomics is to elucidate the factors that explain the variance of gene structures within and between species. It has been hypothesized, for example, that differential GC content of exons and introns within regions of low GC content in the genomes of mammals constitutes a marker for exon recognition during splicing and is thus a factor that stabilizes exon-intron boundaries [3, 4]. As further examples, hypotheses on the evolution of gene structure organization state that introns are generated by the insertion of non-autonomous DNA-transposons [5] or, in birds, that selection on intron size is driven by the evolution of powered flight [6]. Such hypotheses and observations are based on the structural description of protein-coding gene repertoires. These repertoires are typically derived from automated annotations, with only a fraction of the gene models having been refined by manual annotation and curation.

Since the 1980s, procedures for automated gene structure prediction have been developed and continuously improved (reviewed by, for example, [7–9]), but they are still not error free [10–12]. The most commonly encountered errors are false positive and false negative identifications of protein-coding nucleotide sequences [13, 14], non-coding nucleotide sequence retention in coding exons [15], wrong exon and gene boundaries [14, 16], and fragmented or merged gene models [15, 17, 18]. With increasing size and structural complexity (i.e., increasing exon count) of genes, annotation errors are increasingly likely to occur and thus impair the accuracy of automated annotations [16, 19, 20]. Furthermore, gene density can influence annotation results [21]. For example, during the automated annotation of the large, ‘gene-sparse’ genome of the bug *Oncopeltus fasciatus*, many genes were wrongly split across multiple models (“the number of genes resulting from a merged CDS action is far greater than the number of gene models resulting from split CDS actions” [19], Supplement p. 27, and references therein]). In contrast, the ‘gene-dense’ genome of the centipede *Strigamia maritima* showed “in a significant number of cases, [that] the automated annotation [...] fused adjacent genes, largely on the basis of confounding RNASeq [sic] evidence” [22], Supplement p. 3].

The severity of the aforementioned annotation errors is influenced by assembly quality [2, 20, 23], which in turn is influenced by genome size and repeat content [24, 25]. The results of automated annotation additionally depend on whether or not extrinsic evidence (i.e., alignments of homologous or orthologous sequences from other species) is used for gene sequence delineation. Algorithms that incorporate extrinsic evidence will likely more reliably predict genes with conserved coding sequence [26]. However, genes that do not resemble the provided extrinsic evidence — being, for example, taxon-specific — could be missed during automatic annotation [27]. Thus, annotation results depend on the availability and quality of evidence to support the annotation procedure [28, 29]. Despite these caveats, advantages of automated gene annotation include the speed and ease of application to (multiple) genome assemblies as well as reproducibility due to the application of explicit algorithms. With an expected average of 21,500 protein-coding genes in a eukaryotic genome [30], the automated approach is the method of choice to comprehensively annotate genes in a given genome, despite the risk of erroneous models. In comparative analyses, erroneous models have been held responsible for (i) false positive and false negative detection of clade-specific genes [31, 32], (ii) inference of incorrect gene copy numbers [13], (iii) biased correlations between biological traits [32], and (iv) misleading functional annotations [33]. Errors in the annotation of protein-coding genes have been shown to mislead the analysis of gene family evolution [13], protein innovation rates [31], and the interpretation of gene function [33].

Automatically generated gene models can be reviewed and corrected individually in a subsequent process termed manual annotation or manual curation. Although often used interchangeably, here we use “manual annotation” to refer to adding or correcting gene model structures, and “manual curation” to imply additionally associating gene models with names, symbols, descriptions, or putative functions through examining experimental data and by considering information from the literature. Note that there are alternative understandings of these terms (e.g., within the i5k community [37]), with “annotation” considered the de novo creation of a model and “curation” encompassing review and editing of an existing model, considering all available structural and functional information. Annotation and curation efforts have proven to be most rewarding. For example, manual annotation helped to annotate nested and overlapping genes in the fruit fly [10], doubled the number of identified ionotropic receptors in two mosquitoes [34], and led to the discovery of elevated non-canonical splice site usage in a copepod [35]. To some extent, these examples represent ‘special cases’ that required manual annotation: the failure of the automated annotation strategies could be explained by gene structural complexity, high levels of gene sequence divergence, or rare deviations from canonical gene features. Beyond such cases, and beyond individual genes, it remains unclear whether manual annotation impacts genome-wide distributions of gene model structural properties, and if so, how and how much? If manual annotation does have a substantial effect, then comparing genome-wide trends in gene structural properties among different species or lineages would need to control for these effects. On the other hand, if the genome-wide effects of manual annotation are negligible, then comparative analyses can confidently employ automatically inferred gene models to characterize true biological/evolutionary differences in gene structural properties. Our thorough search for published assessments of the extent to which manual annotation affects genome-wide trends of gene structural properties in comparatives analyses revealed only one highly relevant but outdated article [10]. Results of such studies are, however, likely of broader interest, given that gene structural properties of both automatically inferred and manually annotated gene models are frequently compared across species.

To address this issue, here we compare automatically inferred and manually annotated gene models with respect to five structural properties, namely transcript, protein, intron, and exon lengths as well as exon count. Our data for these comparisons comprises the protein-coding gene sets of seven insect species that represent taxonomically distant clades (last common ancestor ca. 370 million years ago [36]) and whose genomes differ in size and assembly quality from each other (Table 1, Additional file 1: Table ST1; *Anoplophora glabripennis* and *Leptinotarsa decemlineata* [Coleoptera], *Cimex lectularius* and *Oncopeltus fasciatus* [Hemiptera], *Athalia rosae* and *Orussus abietinus* [Hymenoptera], and *Frankliniella occidentalis* [Thysanoptera]). These genomes were processed in the context of the i5K pilot project for insect and arthropod genome sequencing [37] with an identical set of methodologies [39] (i.e., sequenced, assembled, and protein-coding genes annotated with the MAKER2 pipeline [38]). Additionally, substantial subsets of the automatically annotated gene models, hereafter referred to as ‘predecessors’, were manually annotated in all seven species (3.5–6.9% of the original gene models, > 650 models per species, Table 1). Manual annotation also yielded de novo gene models without predecessors (0.4–2.2% of the OGS, 30–381 models, Table 1).

**Table 1:** Summary statistics of the genomes, automatically annotated and manually annotated gene sets, and gene model properties for the seven analyzed species

		Holometabolous				Hemimetabolous		
		Coleoptera		Hymenoptera		Hemiptera		Thysanoptera
		*Anoplohora glabripennis*	*Leptinotarsa decemlineata*	*Athalia rosae*	*Orussus abietinus*	*Cimex lectularius*	*Oncopeltus fasciatus*	*Frankliniella occidentalis*
	Assembly size [Mbp] (% determined nucleotides)	707.7 (85.1)	1170.2 (58.0)	163.8 (95.7)	201.2 (92.7)	650.5 (79.0)	1098.7 (70.4)	415.8 (63.4)
	AUTO	22,253	24,732	11,956	10,966	14,085	19,587	18,021
	OGS	22,035	24,671	11,894	10,959	13,953	19,615	17,553
	AUTO-	749	972	805	659	795	1013	1118
	SUB							
	AUTO-SUB % of AUTO	3.4	3.9	6.7	6.0	5.6	5.2	6.2
	MAN-SUB	770	933	825	670	778	945	1127
	MAN-SUB % of OGS	3.5	3.8	6.9	6.1	5.6	4.8	6.4
	MAN-ADD	216	98	50	30	221	161	381
	MAN-ADD % of OGS	1.0	0.4	0.4	0.3	1.6	0.8	2.2
Median transcript length [bp]	AUTO-SUB	6183	8562.5	4340	5200	4362	9324	5001.5
	MAN-SUB	5789.5	9280	3208	3996	4360	11,244	4064
Median protein length [aa]	AUTO-SUB	358	255	445	430	358	257	419.5
	MAN-SUB	389	300	423	419	372.5	320	419
Median exon count p.t.	AUTO-SUB	4	4	6	5	5	4	6
	MAN-SUB	4	4	5	5.5	5	4	6
Median median exon length p.t. [bp]	AUTO-SUB	1210	984	2220	2151	1200	1086	1807.5
	MAN-SUB	1345.5	1127	1786	1828	1194.5	1347	1755
Median median intron length p.t. [bp]	AUTO-SUB	354.75	1192	107.5	1278.25	75	126.75	108
	MAN-SUB	359	1363	100.5	1434	74	123	100.75

Summary statistics on assemblies and manual annotation actions for each species and selected set-wide property values of MAN-SUB and AUTO-SUB

*aa* amino acids, *bp* base pairs, *det. Nucs*. determined nucleotides (i.e., not N), *Mbp* mega base pairs, *OGS* official gene set, *p.t.*: per transcript

Using the above data, we assessed to what extent the previously mentioned five gene structural properties changed due to manual annotation (relative to the automatically inferred predecessor models). We furthermore studied whether previously reported correlative trends of structural features are detectable when analyzing automatic predictions and manual annotations. Specifically, we tested whether genome size correlates negatively with (i) the coding proportion of the genome (i.e., here total length of all exons relative to genome size; see Methods) [30], and whether genome size correlated positively with (ii) the intronic proportion of the genome [20, 30] and (iii) gene count [30]. We also examined whether we are able to confirm a negative correlation between exon/intron count per gene and (iv) exon/intron length and [40] (v) the GC content of the exons/introns [40].

## Results

### Structural properties of manually annotated gene models and their predecessors

We assessed five structural properties of protein-coding genes when comparing automatically generated and manually annotated gene models: (i) unspliced transcript (pre-mRNA) length, (ii) protein length, (iii) exon count per transcript, as well as (iv) median exon and (v) median intron length per transcript. These properties were analyzed in two gene sets: (1) the full set of automatically generated gene models (AUTO) and (2) the full official gene set (OGS; non-redundant merge of gene models that were manually annotated or added and automatically generated models). We additionally studied these gene structural properties in three subsets of gene models: (3) all manually annotated gene models (MAN-SUB), (4) all automatically generated predecessors of the manually annotated gene models (AUTO-SUB), and (5) all manually added de novo gene models (MAN-ADD) (counts of gene models per set and species are given in Table 1).

We first asked how well the subsets reflect the structural properties of the full sets. Thus, we compared the gene set AUTO-SUB with the gene set AUTO and the gene set MAN-SUB with the gene set OGS (Additional file 2: Figure SF1). Most distributions and central tendencies of structural properties differ between subsets and full sets (p adj. ≤ 0.05 in 57.1% of AUTO vs. AUTO-SUB comparisons and in 71.4% of OGS vs. MAN-SUB comparisons with Bonferroni-corrected two-sample Kolmogorov-Smirnov [KS-test] and/or two-sample Wilcoxon [W-test] tests, Additional file 1: Table ST3). Furthermore, we employed a jackknife resampling approach to establish confidence intervals of correlation coefficients to assess how well trends observed in our subsets represent those found in the full sets across a total of 28 comparisons (seven species, four correlations: median exon GC content vs. exon count, median exon length vs. exon count, median intron GC content vs. intron count, and median intron length vs. intron count). We found that the correlation coefficient of the AUTO-SUB subset lay outside of the interval established by resampling from the AUTO set in 20 of the 28 analyzed correlations. Likewise, we found that the correlation coefficient of the MAN-SUB subset lay outside the interval established by resampling from the OGS set in 18 of the 28 analyzed correlations (Additional file 2: Figure SF5, Additional File 1: Table ST4). These deviations can be interpreted as instances in which the subset does not reflect the respective full set regarding a certain combination of parameters. For example, in *A. rosae*, the interval established for the correlation coefficient of exon count compared to GC content drawn from the OGS is r = − 0.04–0.18, with the value of the OGS itself meeting the median (r = 0.06), while the value of the MAN-SUB set (r = − 0.15) is lower than the interval minimum (i.e., r = − 0.04) (Additional file 1: Table ST4). This suggests that models chosen for manual annotation are not in themselves a representative subset of all protein-coding gene models (models are not selected randomly, as researchers usually focus on particular gene families of interest, discussed below). Nonetheless, our primary concern was whether the act of manual annotation appreciably alters the structural properties of the chosen models.

**Fig. 1:**
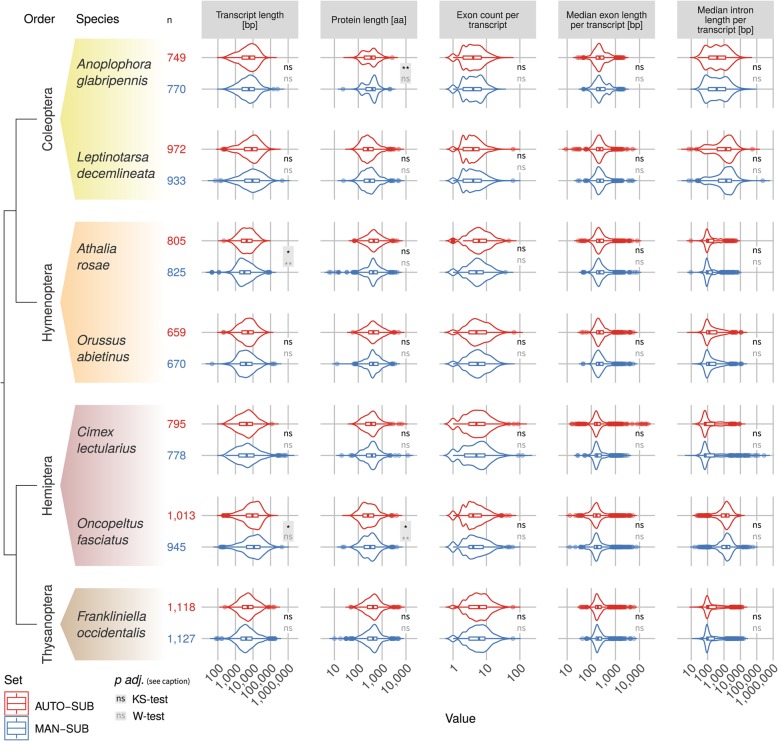
Comparison of property distributions: AUTO-SUB vs. MAN-SUB. Distributions (violin plots) of five gene structure properties per genome (semi-logarithmic) comparing AUTO-SUB (top, red) and MAN-SUB (bottom, blue): unspliced transcript length [bp], protein length [aa], exon count p.t., median exon length p.t. [bp], median intron length p.t. [bp] in facet columns. Additionally, box plots indicate the quartiles of the data distributions; lower and upper hinges correspond to the first and third quartiles. Samples sizes are given (n, AUTO-SUB: red, MAN-SUB: blue). Values are derived from the longest predicted transcript per gene. Adjusted *p*-values of Bonferroni-corrected two-sample Kolmogorov-Smirnov (KS) tests (black) and two-sample Wilcoxon (W) tests (gray) are indicated for each combination of AUTO-SUB vs. MAN-SUB (per species and property) and displayed with gray background if one of these is significant. ns: not significant. Facet rows contain seven species (*Anoplophora glabripennis* [Coleoptera], *Athalia rosae* [Hymenoptera], *Cimex lectularius* [Hemiptera], *Frankliniella occidentalis* [Thysanoptera], *Leptinotarsa decemlineata* [Coleoptera], *Oncopeltus fasciatus* [Hemiptera], *Orussus abietinus* [Hymenoptera]). Taxonomic orders are color-coded, color codes represent the insect orders Coleoptera (yellow), Hymenoptera (orange), Hemiptera (burgundy), and Thysanoptera (brown). The left side tree illustrates the order-level phylogenetic relationships (after [36])

In fact, in comparing the subset-wide distributions of structural properties of AUTO-SUB and MAN-SUB with each other (comprising 3.5–6.9% of AUTO/OGS in each species), we find significant differences in the analyzed gene structural parameters for only four parameters in three species (out of 35 assessments): (i) *A. glabripennis*: protein length (KS-test: p adj. = 0.007), (ii) *A. rosae*: transcript length (KS-test: p adj. = 0.031, W-test: p adj. = 0.008), and (iii) *O. fasciatus*: protein length (KS-test: p adj. = 0.011, W-test: p adj. = 0.003) and transcript length (KS-test: p adj. = 0.021) (Fig. 1; Additional file 1: Table ST3).

**Fig. 2:**
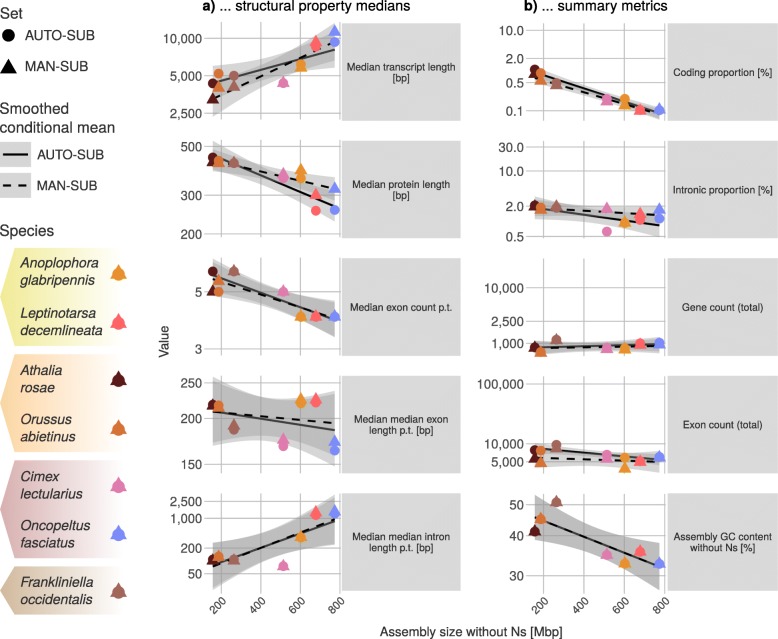
Comparison of AUTO-SUB and MAN-SUB subsets regarding correlations of... **a**) ... structural property medians (in rows from top to bottom): median unspliced transcript length [bp], median protein length [aa], median exon count p.t., median median exon length p.t. [bp], and median median intron length p.t. [bp] of AUTO-SUB (circles) and MAN-SUB (triangles) (semi-logarithmic). Notably, manual annotation of genes in two genomes with the largest assemblies (*L. decemlineata*, 1170 Mbp and *O. fasciatus*, 1099 Mbp) led to an increase (from AUTO-SUB to MAN-SUB, W-test) of the median transcript length (*L. decemlineata*: + 717.5 bp, p adj. = 1; *O. fasciatus*: + 1920 bp, p adj. = 0.07) and of the median protein length (*L. decemlineata*: + 45 aa, p adj. = 0.28; *O. fasciatus*: + 63 aa, p adj. = 0.003). In the three species with the smallest genome sizes in our sample (*A. rosae*, 163.8 Mbp; *O. abietinus*, 201.2 Mbp; *F. occidentalis*, 415.8 Mbp), manual annotation resulted in slight decreases of median transcript length (*A. rosae*: − 1132 bp, p adj. = 0.008; *O. abietinus*: − 1204 bp, p adj. = 1; *F. occidentalis*: − 937.5 bp, p adj. = 1) and median protein length (*A. rosae*: − 21 aa, p adj. = 1; *O. abietinus*: − 11 aa, p adj. = 1; *F. occidentalis*: − 0.5 aa, p adj. = 1). The two species with intermediate assembly sizes (*A. glabripennis*, 707.7 Mbp; *C. lectularius*, 650.5 Mbp), manual annotation resulted in a negligible decrease in median transcript length (*A. glabripennis*: − 393.5, p adj. = 1; *C. lectularius*: − 2 bp, p adj. = 1) and a slight increase in median protein length (*A. glabripennis*: + 31, p adj. = 1; *C. lectularius*: + 14.5 aa, p adj. = 1). **b**) … summary metrics (in rows from top to bottom): coding proportion [%] (i.e., the summed lengths of all exonic sequences in the annotation in relation to genome size), intronic proportion [%], total gene count, total exon count, and assembly GC content without ambiguity [%] of AUTO-SUB (circles) and MAN-SUB (triangles) (semi-logarithmic). Values are derived from the longest predicted transcript per gene. Line types indicate the smoothed conditional mean for AUTO-SUB (solid) and MAN-SUB (dashed). aa: amino acids; bp: base pairs; Mbp: mega base pairs; p.t.: per transcript; W-test: Bonferroni-corrected two-sample Wilcoxon test

Complementing these statistical tests, when regarding the subset-wide medians of AUTO-SUB and MAN-SUB (Fig. 2a), we distinguish three species groups by assembly size and overall effect direction in terms of how median transcript length and median protein length are affected by manual annotation (Fig. 2a, Table 1; Additional file 1: Table ST3). These are: (i) two species with large genomes and increased transcript and protein length after manual annotation (i.e., *L. decemlineata* and *O. fasciatus*, with genome sizes of ca. 1.1 Gbp), (ii) three species with small genomes and decreasing tendencies (the hymenopteran and thysanopteran species, with genomes ranging 164–416 Mbp), and (iii) two species with intermediate-sized genomes and mixed tendencies of minor transcript reduction yet slightly increased protein lengths after manual annotation (*A. glabripennis*, 707.7 Mbp; *C. lectularius*, 650.5 Mbp). To some extent, these tendencies with respect to genome size corroborate the reported species-specific assessments noted above on the effect of gene density on automatic model correctness [19, 22].

Lastly, we evaluate the de novo models in the minor MAN-ADD subsets, which contain 30–381 gene models per species. Strikingly, more than 80% of the gene structure property distributions of MAN-ADD gene models differ significantly (KS-tests and/or W-tests: p adj. ≤ 0.05) from the property distributions of the gene models in the gene sets AUTO, AUTO-SUB, OGS, and MAN-SUB (Additional file 1: Table ST3, Additional file 2: Figures SF1 and SF3). Additionally, the correlation coefficient of the MAN-ADD subset lay outside of the interval established by resampling from the OGS set in 22 of the 28 analyzed correlations (Additional file 1: Table ST4, Additional file 2: Figure SF4). To further explore these differences, we exemplarily analyzed MAN-ADD of *O. fasciatus*, where 70.2% of the subset’s gene models specifically code for cuticle proteins and chemoreceptors (primarily gustatory receptors). Thus, property distributions of MAN-ADD are mainly governed by the specific properties of these gene families; however, we do not go into detail here due to small sample sizes (Additional file 3: Note S2, Additional file 1: Table ST7, Additional file 2: Figure SF5).

### Sets of predecessors and manually annotated gene models agree when analyzing reported correlations

**Fig. 3:**
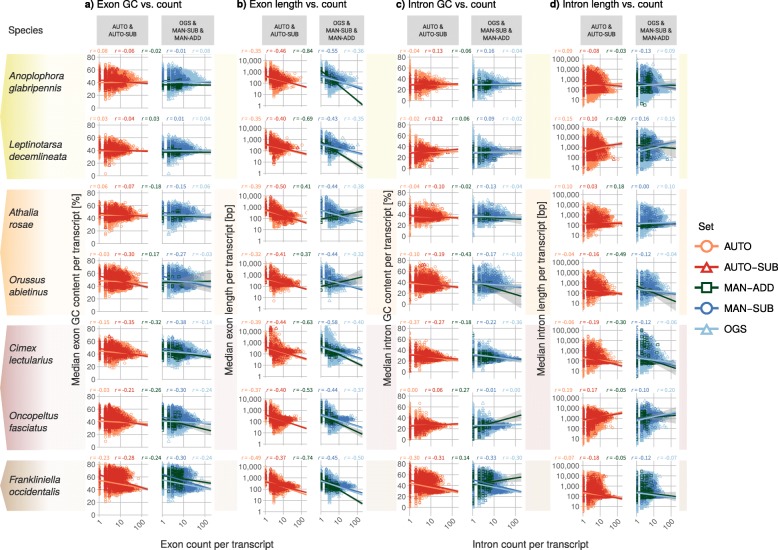
Selected gene structure property correlations in all sets. **a**) Exon GC vs. count: Logarithmic display of median exon GC content [%] vs. exon count per transcript. **b**) Exon length vs. count: Semi-logarithmic display of median exon length [bp] vs. exon count per transcript. **c**) Intron GC vs. count Logarithmic display of median intron GC content [%] vs. intron count per transcript per transcript. **d**) Intron length vs. count: Semi-logarithmic display of median intron length [bp] vs. intron count per transcript. Facet columns show the two automatically generated sets (AUTO & AUTO-SUB; left) and three OGS-based sets (OGS & MAN-SUB & MAN-ADD; right). Values are given for the longest transcript per gene. Spearman’s rank correlation coefficients (r) of each property combination are given above each pair of plots (AUTO: orange, AUTO-SUB: red, MAN-ADD: dark green, MAN-SUB: dark blue, OGS: light blue). Facet rows show the seven species (*Anoplophora glabripennis* [Coleoptera], *Athalia rosae* [Hymenoptera], *Frankliniella occidentalis* [Thysanoptera], *Leptinotarsa decemlineata* [Coleoptera], *Oncopeltus fasciatus* [Hemiptera], *Orussus abietinus* [Hymenoptera]) with color coding according to Fig. 1

Having established that manual annotation does not greatly affect gene structural properties in themselves, we next assessed how the AUTO-SUB and MAN-SUB gene subsets compare for correlations of genome size and GC content with various structural properties. In only 2 of 28 comparisons (seven species and four property combinations, as above) did we observe a directional change in correlation coefficients from AUTO-SUB to MAN-SUB, with absolute differences of 0.05 and 0.08, respectively (Additional file 1: Table ST4b). Thus, we find almost no differences between correlational trends when comparing structural parameters of genes in the gene subset AUTO-SUB (Fig. 3, left columns) with those of genes in the subset MAN-SUB (Fig. 3, right columns).

Our datasets also provide the opportunity to assess insect species for previously reported correlations of genome size with coding proportion, gene count, and intronic proportion, as described by [20, 30]. Note that due to the low sample size of seven species (a necessary constraint for ensuring common methodology across species and a reasonably high proportion of manually annotated gene models), we subsequently present only descriptive statistics when assessing correlative trends.

Our results are in agreement with the finding [30] that the coding proportion of the genomes is negatively correlated with the genome size and that the total gene count increases with genome size (Fig. 2b, Table 1). In contrast, reports for other correlations [20, 30] are not borne out by our insect data. Specifically, we see no or only a weakly negative correlation between the intronic proportion of a genome and genome size (Fig. 2b, Table 1). We found these trends irrespective of whether we compared the gene set AUTO with the gene set OGS or whether we compare the gene subset MAN-SUB with the gene subset AUTO-SUB (Additional file 2: Figure SF2).

In line with previous results [40], we do find a negative correlation between exon/intron count and median GC content of exons/introns in 21 of 28 comparisons (the seven species and four gene sets: AUTO, AUTO-SUB, OGS, and MAN-SUB; Additional file 1: Table ST4b). Notably, complex gene models (> 50 exons) are less variable in the GC content of their introns (ca. 20–45%) than less complex models (ca. 10–60%, Fig. 3a, c); this relationship does not seem to be influenced by genomic transcript length (data not shown). *F. occidentalis* conspicuously has two classes of complex gene models with low (as in the other species, ca. 20–40%) and high (ca. 0–60%) GC content variability in introns (Fig. 3c). Gene models with more than ten exons appear to be restricted to a certain median exon length class (ca 190 bp); this coincides with a negative correlation of exon count and median exon length (28 of 28 comparisons; Fig. 3b, Additional file 1: Table ST4b), as was also reported by [19, 40]. In contrast to the report by Zhu et al. [40], we observe mixed trends (among species, not among sets except within *A. glabripennis*) regarding the correlation of intron count and median intron length (Fig. 3d): some species exhibit a positive correlation (*A. rosae*, *L. decemlineata*, *O. fasciatus*) while others show a negative correlation (*C. lectularius*, *F. occidentalis*, *O. abietinus*) in the four (sub)sets (Additional file 1: Table ST4). Thus, while certain correlations among genome and gene structural properties appear to also apply in insects, other correlations vary across taxa.

## Discussion

### Limitations of the present study

The quality of automatic and manual annotations is strongly impacted by genome assembly quality and by the availability of extrinsic evidence such as orthologous sequences from closely related species, and RNA-seq data [6, 20]. The impact of these factors on the correctness of gene models is beyond the scope of our study. Assessing the biological correctness of gene models remains difficult without a validated benchmark set [16, 41] or appropriate quality metrics. The BUSCO quality metric [42] indeed makes a distinction between complete and partial orthologs, but this approach is limited to the subset of highly conserved protein-coding genes. However, we ensured comparability between genome assemblies and annotations by a conservative selection of species. The genomes and gene sets of the selected species have been inferred with the same wet lab and bioinformatic approaches [39]. Extending the taxonomic sampling at the time of data collection would have resulted in jeopardizing this methodological consistency and comparability. Thus, we analyzed the largest possible set of i5K species in terms of availability of gene sets before and after manual annotation at the time of data collection. All annotations and the derived statistics are based on de novo assemblies resulting from short-read sequencing paired and mate pair libraries, which are inherently fragmented. It remains to be tested whether the same conclusions can be drawn regarding the suitability of automatically inferred genes sets for comparing gene structural parameters when analyzing the gene sets of genomes assembled to higher quality (as reviewed by [20]). Similarly, our study based on 3.5–7% of protein-coding genes being manually annotated represents an assumed extrapolation whose conclusions could change once all genes would be manually annotated.

### Repertoire-wide gene structure assessments can rely on automatically predicted gene models

The finding that the analyzed subsets (i.e., AUTO-SUB and MAN-SUB) do not fully reflect the property distributions of the respective full sets (AUTO, OGS) may give rise to concern whether generalizations are justified. However, we did not find a bias in either subset towards a certain combination of structural properties. Thus, at least the diversity of gene structures of the full sets appears to be reflected in the subsets.

We find that the distributions, gene set-wide medians, and correlative trends of gene structure properties of AUTO-SUB are very similar to that of MAN-SUB (Figs. 1, 2 and 3). The analyses comparing AUTO-SUB and MAN-SUB with the respective full sets were conducted excluding MAN-ADD models, since these are added by curators in the absence of an automatically predicted predecessor. However, the hypothesis that automatically predicted gene models suffice as the basis for comparative analyses of large-scale gene structural properties can only be substantiated if the fraction of missing models is comparatively small. In each of seven species analyzed by us, 2.1% or fewer of the OGS gene models had been added manually (Table 1). However, de novo genes models make up a larger fraction of genes handled by curators (4.3–25.3%; i.e., MAN-ADD as fraction of MAN-SUB + MAN-ADD; Additional file 1: Table ST2). MAN-ADD structural properties differ strongly from the remaining four (sub)sets of gene models. These differences likely reflect the highly biased selection of gene classes for manual annotation based on the research interests of the curators, which we address here for cuticle structural proteins and chemoreceptors as exemplar classes. In particular, chemoreceptor genes are notoriously difficult to automatically predict (rapidly evolving genes with low expression levels of transcripts, (e.g., [19]). Thus, they are frequently added de novo, as found in the annotation of the *O. fasciatus* genome [19] (Additional file 3: Note S2), and gene structural property distributions may be strongly governed by distinct gene families (Additional file 2: Figure SF5). Although de novo gene models appear to be heavily biased in terms of their structure (Additional file 2: Figures SF1 and SF3), we expect that overall trends and distributions are only negligibly affected by them due to their small overall count.

### Predecessors and manually annotated gene models agree on correlative trends of gene structure

Given the general agreement of gene structure properties between AUTO-SUB and MAN-SUB gene models, we tested whether or not we also find an agreement between automatic and manual annotation when investigating large-scale trends. Specifically, we investigated whether we could confirm previously reported gene structure trends in relation to genome size.

Our results are in line with previous reports [20, 30] regarding the negative correlation between coding proportion and genome size (Fig. 2b). This result is in line with the hypothesis that genome size is mainly driven by repeat content rather than by gene count [24]. On the other hand, we do not recover the previously reported [20, 30] positive correlation between intronic proportion and genome size. Since previous studies analyzed data from four [30] and six [20] phyla of Eukaryota with insects being represented by only few species, we might observe an insect-specific pattern. However, further studies are necessary to verify that this trend is not caused by small sample size or genome quality. If a different pattern of intron evolution can be corroborated in insects, assumptions on general genome evolution would have to be re-evaluated. It was indeed recently shown that there is evidence for a positive correlation of genome size and intron count in insects [19] and for highly dynamic intron evolution in a phytoseiid predatory mite [43]. On the other hand, short read sequencing technologies for genome assembly may limit sensitivity for detecting this correlation, as long introns may fail to be fully assembled.

A negative correlation of exon count and exon length, as consistently found in our data (Fig. 3b), has been reported not only in the genomes of human and rice [40], but also in that of insects [19]. Furthermore, we find a negative correlation of exon/intron count and respective GC content as well as an apparent constraint of complex gene models to a medium GC content, especially in introns (Fig. 3a, c), as previously reported [40]. However, we recover the reported [40] negative correlation of intron count and length only in three (*O. abietinus*, *C. lectularius*, *F. occidentalis*) of the seven species in all (sub)sets, while in *A. glabripennis* we see the trend only in the full sets (AUTO and OGS) (Fig. 3d, Additional file 2: Figure SF3). These results could point towards insect-specific and intron-specific peculiarities in the evolution of gene structure [19, 43]. The vertebrate-biased taxon sample used by Zhu et al. [40] (nine vertebrates, two plants, one worm, and one insect) does not allow one to draw conclusions with respect to insects. While an amniote-specific positive correlation of intron and genome size has been shown and discussed in relation to avian powered flight [6], it has yet to be determined whether introns evolve in a manner specific to insects and whether it is affected by other constraints than in amniotes.

## Conclusions

Focusing on a diverse sample of insect genomes, we analyzed whether repertoire-wide distributions of gene structural properties change when automated annotations of protein-coding genes are manually revised. Our results suggest that the influence of manual annotation on the distribution of those properties studied by us is comparatively small, even if individual models may have substantially changed in detail. Thus, our study empirically supports the generally accepted but to date not extensively tested view that automated gene prediction yields reliable gene models. We further conclude that automatically predicted gene models allow the elucidation of commonalities, differences, and driving forces of gene structure evolution: we consistently (with few exceptions) find correlative trends in the analyzed gene structural properties when using either automatically generated or manually annotated models. While manual annotation is fundamentally important to obtain accurate gene models, our results suggest that the insect-specific patterns of gene structure described here can be addressed without the necessity of prior manual annotation when using assemblies and annotations of high quality. Establishing that manual annotation does not substantially impact analyses of genome-wide trends is important for large-scale studies such as carried out within the i5K project [39], where manual annotation of the included species’ gene sets varies from none to extensive.

## Methods

### Data sample

We analyzed annotations and assemblies of seven insect species of four orders (Coleoptera: *Anoplophora glabripennis* [44], *Leptinotarsa decemlineata* [45]; Hemiptera: *Cimex lectularius* [46], *Oncopeltus fasciatus* [19]; Hymenoptera: *Athalia rosae*, *Orussus abietinus* [47]; Thysanoptera: *Frankliniella occidentalis*) [48] that were sequenced and annotated within the i5k initiative [37]. Additional file 1: Table ST1 lists the sources of primary datasets.

### Gene sets

We prepared two sets and three subsets of data from the available annotations produced by the i5k initiative of each species. Firstly, we distinguished the set of all automatic predictions (AUTO) and the final official gene set (OGS) comprising the non-redundant merge of (i) de novo gene models, (ii) manually annotated genes, and (iii) remaining purely automatic gene models. Secondly, we extracted smaller subsets to analyze certain types of annotation in detail: (i) de novo gene models without automatic predecessors (MAN-ADD), (ii) manually annotated gene models that have an automatically predicted predecessor (MAN-SUB), and (iii) the corresponding automatically predicted predecessors to MAN-SUB (AUTO-SUB) (Additional file 3: Note S1).

### Structural property and correlative trend analyses

Structural properties of the predicted protein-coding genes in the respective gene set of each species were inferred with the software COGNATE [49] version 1.01 using the program’s default parameters (COGNATE considers only the longest transcript per gene). Throughout this study, we considered all exons of the longest transcript, also to represent coding sequences. This is due to the fact that UTRs were not consistently annotated (thus, exons and CDSs were identical). All COGNATE results generated for this study (except those of *F. occidentalis*; these are available upon request due to the ongoing publication process) are available from the Dryad repository (datadryad.org): 10.5061/dryad.v50tm7m.

Statistical analyses and visualizations were performed in R [50]. Two-sample Kolmogorov-Smirnov test (KS test, R: ks.test) was used to test for significant differences in structural property distributions between all sets and subsets of each species. Results (across all sets and subsets) were corrected for multiple testing (Bonferroni). In addition, to identify statistical significant differences in central tendencies, each KS-test was supplemented by a two-sample (Mann-Whitney-) Wilcoxon test (Wtest, R: wilcox.test) and results were subjected to multiple test correction (Bonferroni) as well. Both tests address the similarity of distributions, but differ in their sensitivity: the KS test is sensitive to changes in shape, spread, and median between the distributions, while the W test is mostly sensitive to changes in the median.

We used a non-parametric approach to test whether subsets (AUTO-SUB, MAN-SUB, MAN-ADD) can be considered representative for the species-specific sets (AUTO, OGS). To overcome the problem of large size differences between sets and subsets, we used an adaption of the jackknife method (implemented in a custom script available at GitHub, see below). For this, we repeatedly (1000 times) subsampled without replacement 1000 entries (i.e., properties of 1000 gene models) of each set (OGS and AUTO) and calculated Spearman’s rank correlation coefficients of four property combinations: (i) exon count vs. exon length, (ii) exon count vs. exon GC content, (iii) intron count vs. intron length, and (iv) intron count vs. intron GC content. Additionally, the correlation coefficients of the four combinations were calculated for AUTO, OGS, AUTO-SUB, MAN-SUB, and MAN-ADD (Additional file 1: Table ST4). For each species, Spearman’s rank correlation coefficients of the 1000 subsamples are visualized separately for AUTO and OGS, adding the values of the original (sub)sets with a specific color (Additional file 2: Figure SF4).

### Cuticle proteins and chemoreceptors

Intuitively, we expect that fast evolving genes (possibly with rare transcripts) make up a large fraction of genes added de novo during manual annotation. Obvious candidates for such genes are those coding for cuticle proteins (CPs) and chemoreceptors (CRs) [e.g., 5]. The teams of Josh Benoit (Department of Biological Sciences, University of Cincinnati, USA) and Hugh Robertson (Department of Entomology, University of Illinois at Urbana-Champaign, USA) thoroughly manually annotated genes coding for cuticle proteins and chemoreceptors in (at least) *A. glabripennis*, *L. decemlineata*, *C. lectularius*, and *O. fasciatus*. In a small case study, we focused on *O. fasciatus* due to time constraints and compared the manually annotated (i.e., with an automatically predicted predecessor) to added (i.e., de novo) CPs and CRs.

For both CPs and CRs, gene lists were extracted from the *O. fasciatus* OGS v 1.1 according to their annotated name, including information on transcript ID, curation status (manually annotated MAKER model or de novo model), and, for CRs, the chemoreceptor class (gustatory [GR], ionotropic [IR], or odorant [OR] receptors) [Additional file 1: Tables ST5 and ST6]. According to the transcript IDs, COGNATE measurements were extracted for the longest transcript per gene (from the COGNATE output files 07–10). Property distributions are visualized in Additional file 2: Figure SF5.

## Additional files

Additional file 1: **Table ST1.** Data sources and used files: list of publications, download sources, and used files of all seven species; **Table ST2.** Auto vs. manual: counts of gene models that were subjected to manual annotation including non-coding models, as well as of OGS, MAN-ADD, MAN-SUB, AUTO, and AUTO-SUB; **Table ST3.** Tests: results of Bonferroni-corrected two-sample Kolmogorov-Smirnov tests and two-sample Wilcoxon tests of all combinations of the five (sub)sets given for each species and each gene structural property; **Table ST4.** Correlation coefficients: lists for each species and each set (AUTO, OGS, AUTO-SUB, MAN-SUB, MAN-ADD, and resampled samples 1–1000) the Spearman’s rank correlation coefficient of each of the four comparisons; **Table ST5.** Cuticle proteins — all v1.1: list of cuticle protein genes manually added or manually annotated in *Oncopeltus fasciatus*; **Table ST6.** Chemoreceptors — all v1.1: list of chemoreceptor genes manually added or manually annotated in *Oncopeltus fasciatus*; **Table ST7.** CP and CR KS-tests: results of (Bonferroni-corrected) two-sample Kolmogorov-Smirnov tests comparing cuticle protein (CP) and chemoreceptor (CR) gene model structure properties to OGS, MAN-ADD and each other. (XLSX 933 kb)

Additional file 2: **Figure SF1.** Extended version of Fig. 1; **Figure SF2.** Extended version of Fig. 2; **Figure SF3.** Empirical cumulative distribution functions of all sets; **Figure SF4.** Confidence intervals established by jackknifing; **Figure SF5.** Gene structural properties of cuticle proteins (CPs) and chemoreceptors (CRs) of *O. fasciatus*. (PDF 2234 kb)

Additional file 3: **Note S1**. Dataset preparation. **Note S2**. Cuticle proteins and chemoreceptors – Additional results. Captions of supplementary Figures SF1-SF5. (DOCX 27 kb)

## Data Availability

All primary data (assemblies, annotations, RNAseq reads) analyzed during this study are available from repositories as listed in Additional file 1: Table ST1. All datasets except those of /*F. occidentalis*/ generated during this study are available from the Dryad repository (10.5061/dryad.v50tm7m). Custom scripts are available at GitHub (https://github.com/JWilb/Auto-vs-Manual.git).

## Abbreviations

aa: amino acid(s)
bp: base pair(s)
CDS: Coding sequence
DNA: Deoxyribonucleic acid
GFF: General feature format
kb: kilo byte
KS-test: Bonferroni-corrected two-sample Kolmogorov-Smirnov test
Mb: Mega byte
Mbp: Mega base pairs
OGS: Official gene set
ORF: Open reading frame
p.t.: per transcript
RNA: Ribonucleic acid
RNAseq: RNA sequencing/sequences
UTR: Untranslated region
W-test: Bonferroni-corrected two-sample Wilcoxon test
