# Supplementary material for: Repertoire-wide gene structure analyses: a case study comparing automatically predicted and manually annotated gene models
Source: BMC Genomics. 2019 Oct 17;20:753. doi: 10.1186/s12864-019-6064-8 (PMC6798390; doi:10.1186/s12864-019-6064-8)

Supplementary Figure SF1: Gene structural property distributions of all sets (without outliers)

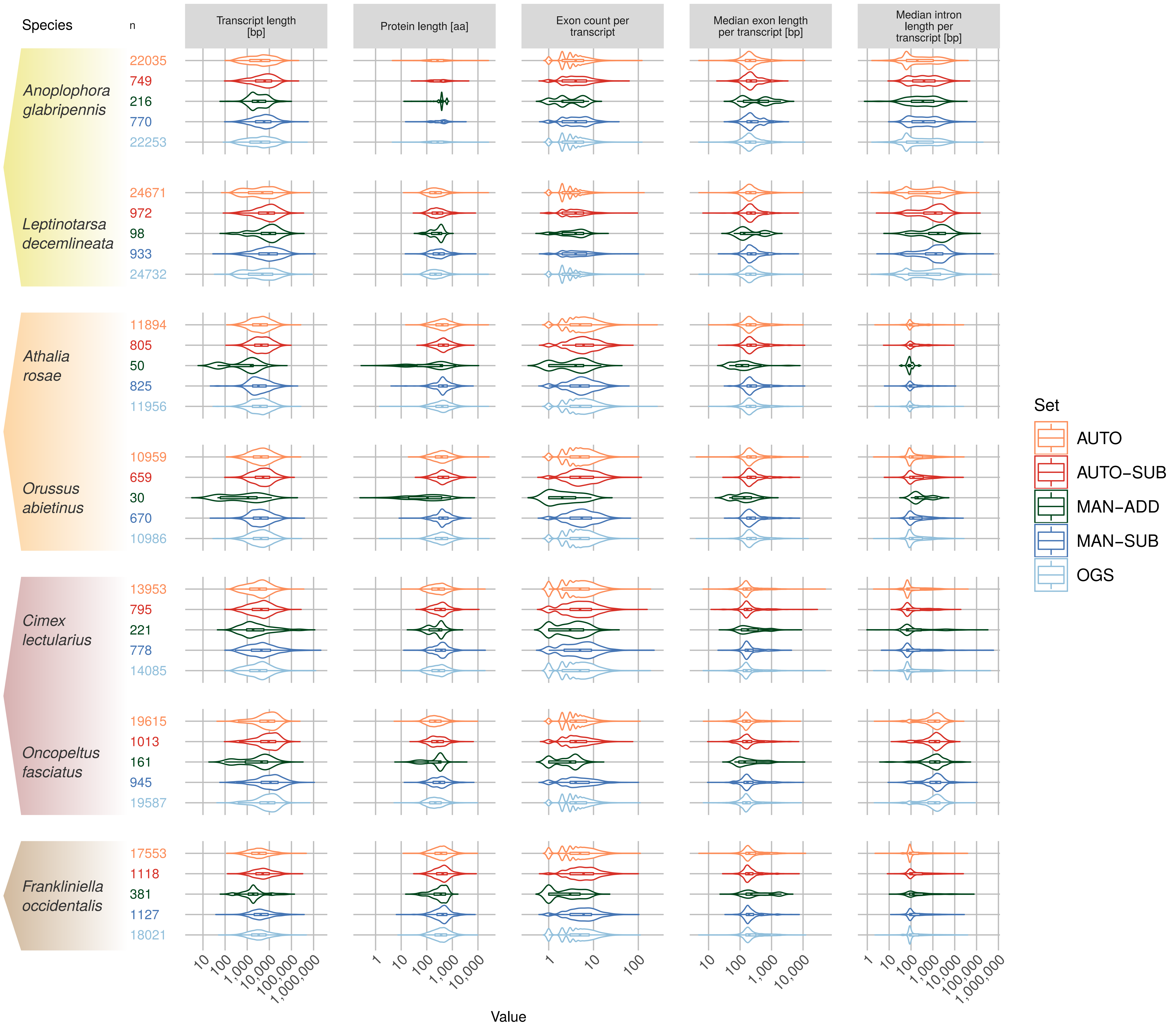

Supplementary Figure SF2: Comparison of all sets regarding correlations of assembly size with...

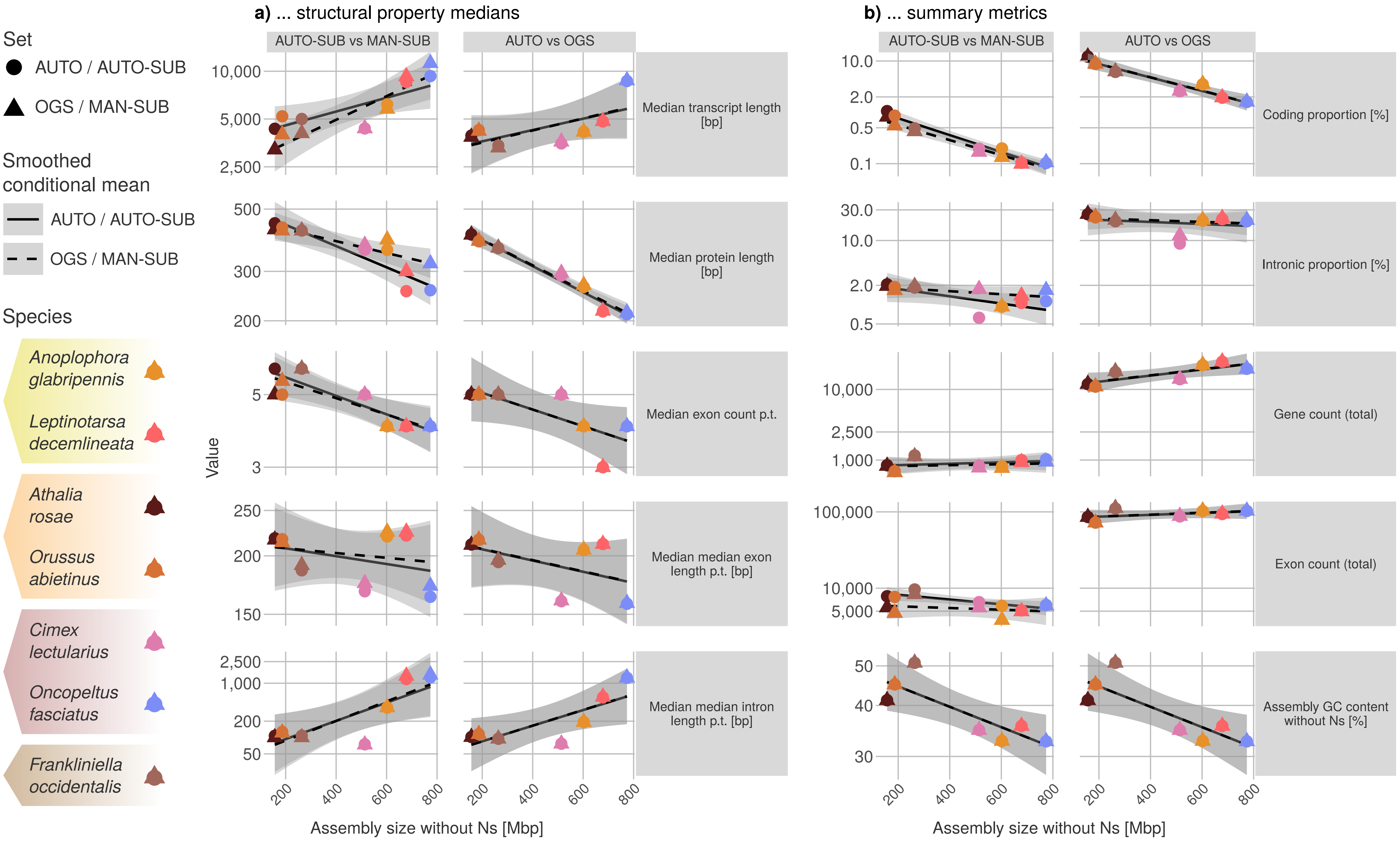

Supplementary Figure SF3: Empirical cumulative distribution functions of all sets

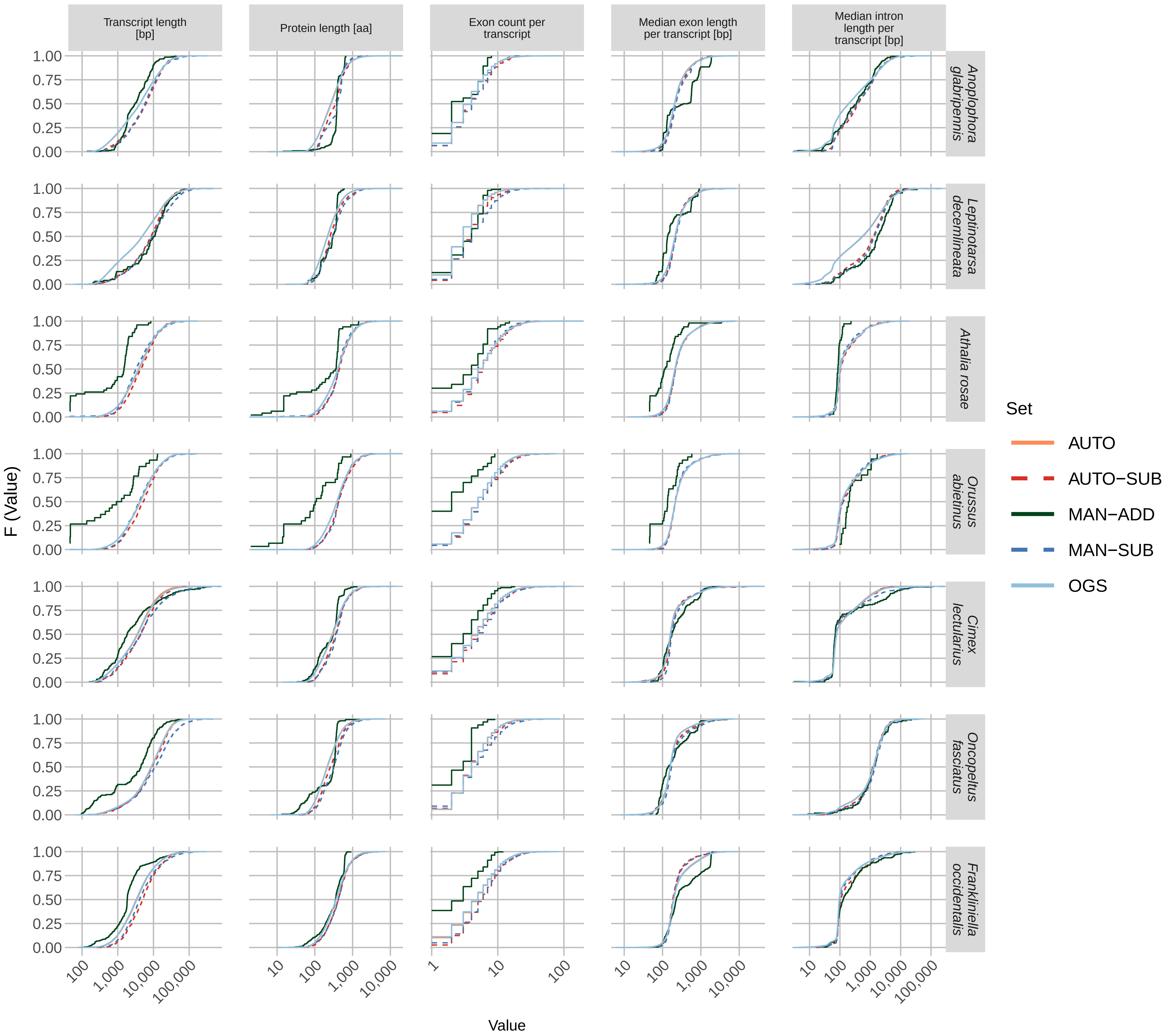

Supplementary Figure SF4: Confidence intervals established by jackknifing

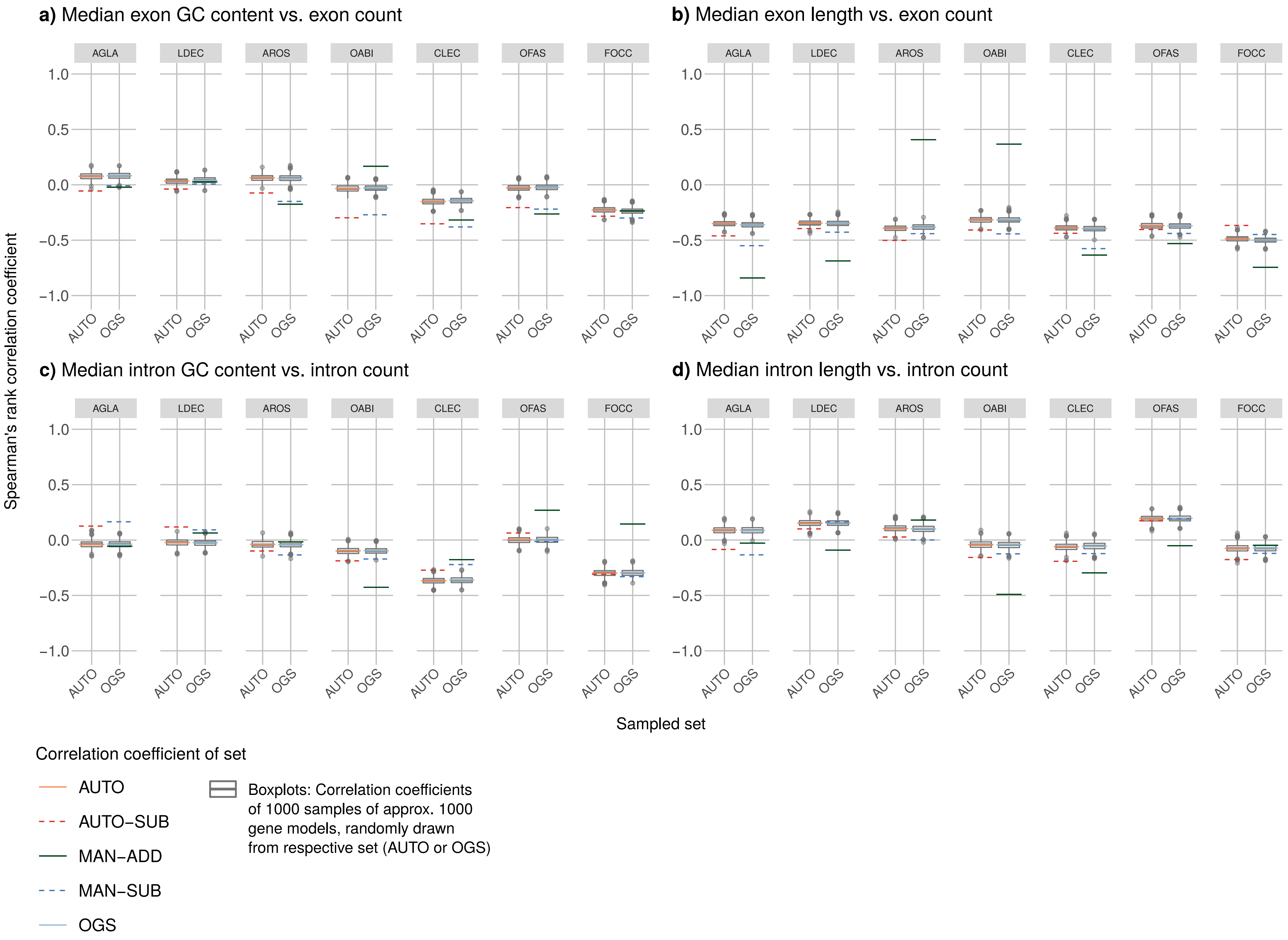

Supplementary Figure SF5: Gene structural property distributions of cuticle proteins (CPs) and chemoreceptors (CRs) of *O. fasciatus*

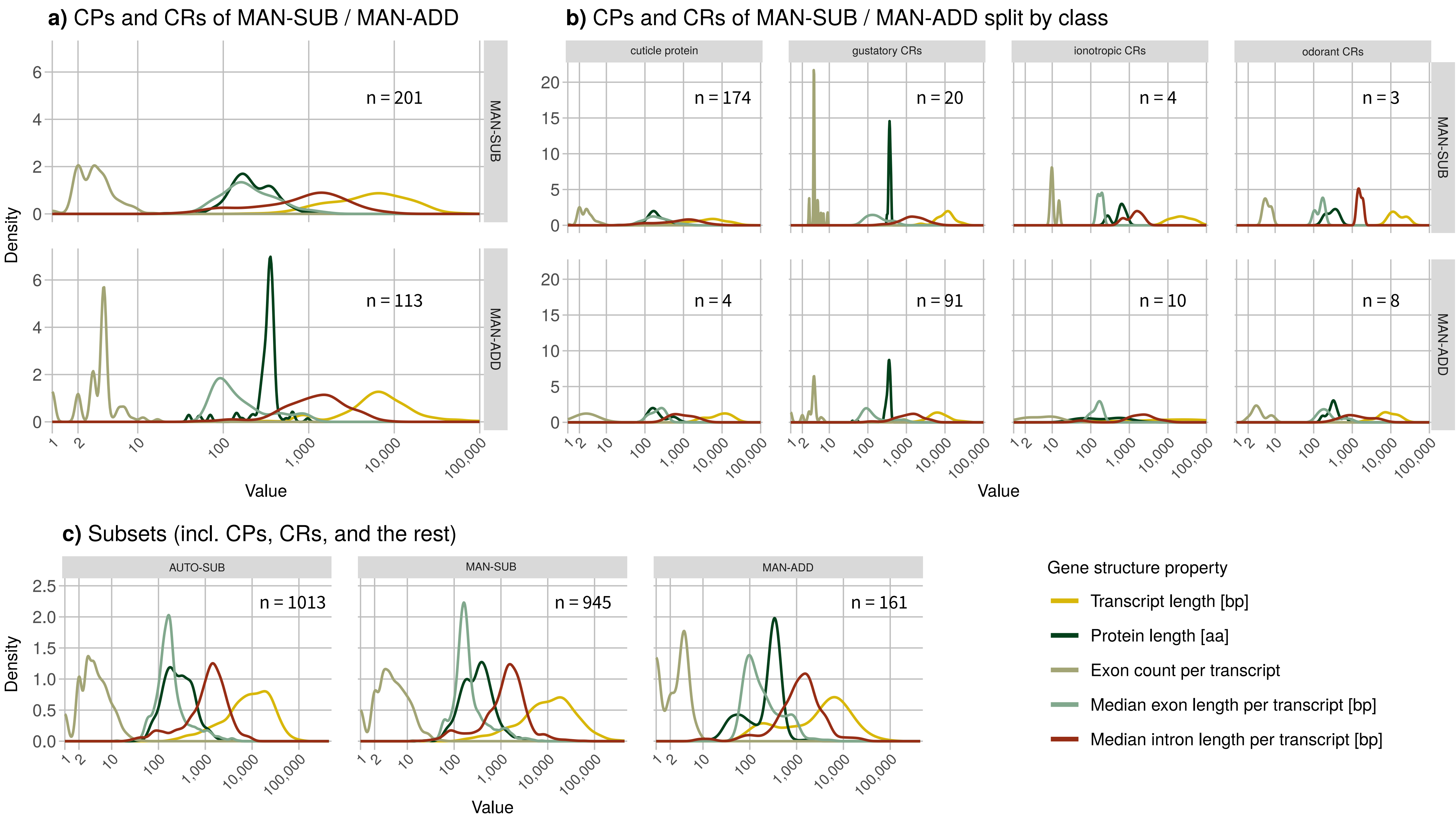

Supplement: Supplementary file 2 — Figure SF1. Extended version of Fig. 1; Figure SF2. Extended version of Fig. 2; Figure SF3. Empirical cumulative distribution functions of all sets; Figure SF4. Confidence intervals established by jackknifing; Figure SF5. Gene structural properties of cuticle proteins (CPs) and chemoreceptors (CRs) of O. fasciatus. (PDF 2234 kb) [file 12864_2019_6064_MOESM2_ESM.pdf]
