# Supplementary material for: Repertoire-wide gene structure analyses: a case study comparing automatically predicted and manually annotated gene models
Source: BMC Genomics. 2019 Oct 17;20:753. doi: 10.1186/s12864-019-6064-8 (PMC6798390; doi:10.1186/s12864-019-6064-8)
Supplement: Supplementary file 3 — Note S1. Dataset preparation. Note S2. Cuticle proteins and chemoreceptors – Additional results. Captions of supplementary Figures SF1-SF5. (DOCX 27 kb) [file 12864_2019_6064_MOESM3_ESM.docx]

**Supplementary Material for**

**Repertoire-wide gene structure analyses: a case study comparing automatically predicted and manually annotated gene models**

Wilbrandt J, Misof B, Panfilio KA, Niehuis O

## **1. Dataset preparation**

Not all automatically generated annotations included UTRs, thus these are not considered in the present study. Prior to set preparation, all non-coding genes (i.e., models without annotated mRNA; 2–339 models in the seven species, **Additional file 1: Table S2**) were removed. Note that due to current limitations of data formats, we count gene parts on different scaffolds as separate genes. Furthermore, all our analyses comparing the location of gene models delineated by the automated annotation procedure or by manual annotation are based on overlaps of whole genes rather than subunits of these. For the comparison of manually annotated gene models with their overlapping predecessors, we excluded deleted models and newly created (*de novo*) models, as they cannot be compared to a successor or predecessor, respectively.

Of the five sets of genes used in our analyses, the first two did not require modification (AUTO and OGS). To generate the three subsets MAN-SUB, AUTO-SUB, and MAN-ADD, the following steps were executed for each species:

- grep manually annotated genes from OGS (indicated as source or tag “ManualCuration”)
- remove from this set all genes without mRNA
- use the resulting file to produce GFF3 output files with:
  - all gene models in the automated annotation (target) that overlap the manually annotated models (query) and lie on the same strand → AUTO-SUB
  - all manually annotated models for which an overlapping predecessor has been found → MAN-SUB
  - manually annotated models without overlapping predecessors (*de novo* models) → MAN-ADD

## **2. Cuticle proteins and chemoreceptors – Additional results**

Of the 945 gene models in the MAN-SUB set, 201 (21.3%) are CPs and CRs. Within this group, 174 cuticle protein genes have been manually annotated, as well as 20 GRs, 4 IRs, and 3 ORs. Of the 161 gene models that have been manually added (MAN-ADD), 113 (70.2%) are CPs and CRs that divide into 4 CPs, 91 GRs, 10 IRs, and 8 ORs. This means that 56% of all *de novo* models belong to the class of gustatory receptors.

Gustatory receptors exhibit a very distinct distribution of protein length and exon count per transcript, namely with extraordinarily narrow peaks, irrespective of their curation status (i.e., manually annotated / added) (**Additional file 2: Figure S5b**). Nonetheless, the distribution of protein length differs significantly between manually annotated and *de novo* GRs, as well as the distribution of transcript length and exon count p.t. (**Additional file 1: Table S5f**). Distribution differences between the other classes (CP, IR, OR) are not very meaningful due to the very small sample size of at least one of the two compared sets in each test.

Comparing the structural parameter distributions of all manually annotated CPs and CRs (n = 201) with those of the OGS, we find significant differences between transcript length, exon count p.t., and median exon length p.t.; all *de novo* CPs and CRs (n = 113) differ significantly from the OGS in all parameter distributions except median intron length p.t. (**Additional file 1: Table S6f**). We find no significant difference between the parameter distributions of CPs (both manually added and added, n = 178) and the MAN-ADD set, which indicates that they are not responsible for the observed differences between the MAN-ADD and MAN-SUB sets.
